# The Role of Calcium in Inflammation-Associated Bone Resorption

**DOI:** 10.3390/biom8030069

**Published:** 2018-08-01

**Authors:** Gordon L. Klein

**Affiliations:** Department of Orthopaedic Surgery and Rehabilitation, University of Texas Medical Branch and Shriners Burns Hospital, Galveston, TX 77555-0165, USA; gklein@utmb.edu; Tel.: +1-409-747-5700; Fax: +1-409-770-6919

**Keywords:** inflammation, bone resorption, calcium-sensing receptor, chemokines, NLRP3 inflammasome

## Abstract

The aim of this mini-review is to discuss the role of calcium in the process of cytokine-mediated bone resorption in an effort to understand the role circulating calcium may play in the resorption of bone. The liberation of calcium and possibly phosphorus and magnesium by bone resorption may sustain and intensify the inflammatory response. We used a burn injury setting in humans and a burn injury model in animals in order to examine the effects on the bone of the systemic inflammatory response and identified the parathyroid calcium-sensing receptor as the mediator of increasing bone resorption, hence higher interleukin (IL)-1 production, and decreasing bone resorption, hence the lowering of circulating ionized calcium concentration. Thus, extracellular calcium, by means of the parathyroid calcium-sensing receptor, is able to modulate inflammation-mediated resorption.

## 1. Introduction

The resorption of bone by inflammatory cytokines is well accepted. Many chronic inflammatory diseases, including rheumatoid arthritis and inflammatory bowel disease, involve resorptive bone loss. In fact, infliximab, a monoclonal antibody to tumor necrosis factor (TNF)-α, reduces bone loss in patients with Crohn’s disease [1]. We know that the mechanism of bone resorption involves stimulation of the ligand of the receptor activator for nuclear transcription factor kappa B (NFΚB), otherwise known as RANK Ligand or RANKL in the osteocyte and the osteoblast by a number of inflammatory cytokines, most notably interleukins (IL)-1 and IL-6, and TNF-α. The ligand RANKL in turn stimulates the differentiation of osteoclasts from their monocyte/macrophage precursors [2]. Thus, we know how cytokines stimulate bone resorption, but we do not know why this occurs. 

Recently, van Niekerk et al. published an opinion piece [3] in which they speculated that the phosphate and magnesium released by bone following resorption might serve as nutrients to activated immune cells. They construct a plausible argument that the phosphate liberated during bone resorption can serve as an energy source for the immune cells as they are being activated. Phosphate is needed to produce the adenosine triphosphate (ATP) that drives the energy reactions from glycolysis, and many of the enzymes engaged in this process will need to undergo phosphorylation to be activated. Moreover, magnesium, which is the third major element stored in bone, can serve as a co-factor in many of the enzymatic reactions to generate the energy needed. In addition, inflammatory cytokines such as IL-1β and IL-6 can upregulate the parathyroid calcium-sensing receptor (CaSR), which reduces the set-point for circulating calcium suppression of parathyroid hormone (PTH) secretion. The result is hypocalcemic hypoparathyroidism, which can preserve body phosphate by preventing phosphate loss in the urine [4,5]. Thus, certain inflammatory cytokines appear to have a dual function. Interleukin-1β and IL-6 can increase bone resorption, thus releasing bone elements such as phosphate into the circulation, and can also upregulate the parathyroid CaSR, which will result in hypoparathyroidism and phosphate retention. While the evidence supporting this hypothesis is currently indirect, the concept of liberation of calcium from bone may play a role in inflammation, as we shall see. This concept is not new and is briefly mentioned by Straub [6] in his review of the pathophysiology of chronic inflammatory disease. However, the idea is not elaborated upon as it is only a small part of his overall scheme.

## 2. Bone Resorption in the Setting of Burn Injury

We can study the role of liberated bone contents, in particular calcium, in the setting of burn injury in which we have not only the clinical experience in humans but also the existence of animal models of burn injury that have contributed to the study of bone resorption in the presence of a robust systemic inflammatory response. Understanding the effects of liberation of calcium and its possible effect on burn injury survival may offer us clues as to why inflammatory bone resorption occurs. 

Specifically, burn injury results in the destruction of the skin barrier to entry of microorganisms into the bloodstream. This damage results in wound infection and, all too often, sepsis, triggering a robust systemic inflammatory response. In children burned over 40% of their total body surface area, circulating concentrations of IL-1β and IL-6 are elevated three-fold and one-hundred-fold, respectively [7]. Serum concentration of TNF-α has also been reported to be elevated in some publications [8]. Both IL-1β [9,10] and IL-6 [11] have been shown to upregulate the parathyroid CaSR. The work of Nielsen et al. [9] and Toribio et al. [10] demonstrated increased CaSR mRNA in bovine [9] and equine [10] parathyroid chief cells when incubated with IL-1β, a response that was blunted when IL-1 receptor antagonist was added to the media. Experiments by Canaff et al. [11] also demonstrated that intraperitoneal injection of IL-6 into rats could upregulate the parathyroid CaSR mRNA and protein in parathyroid, thyroid and kidney cells. CaSR upregulation reduces the set point for circulating calcium suppression of PTH secretion [12]. Thus, a lower circulating calcium concentration, even concentrations in the hypocalcemic range, would be sufficient to reduce PTH secretion. Upregulating mutations of the CaSR have been manifest as hypocalcemia, hypoparathyroidism, and urinary calcium wasting [12]. These are the findings in pediatric burn patients [13]. Furthermore, CaSR upregulation has been confirmed in a sheep model of burn injury as Murphey et al. [14] demonstrated a 50% upregulation of the parathyroid CaSR mRNA 48 h after a 40% body surface area burn under anesthesia. Immunoperoxidase staining of the ovine parathyroid chief cells revealed more membrane-bound CaSR protein in the burned sheep than in the sham-burned controls [14]. Thus, inflammatory cytokines increase bone resorption in pediatric burn patients, liberating more calcium into the circulation and at the same time upregulating the parathyroid CaSR, effectively lowering circulating calcium. The net effect is mild hypocalcemia with urinary calcium excretion approximately twice normal [13]. In contrast, adult burn victims have circulating ionized calcium concentration at or slightly above the upper limits of normal [15,16], and PTH concentrations within the normal range or slightly elevated [15,16], suggesting that they do not experience cytokine-mediated upregulation of the CaSR. Thus, we have established that severe burn injury results in a robust, sustained acute inflammatory response in pediatric patients who respond in part by upregulating their parathyroid CaSR in addition to resorbing bone and liberating calcium.

Previous work done by us [17] on peripheral blood mononuclear cells obtained from normal adults and cultured in media containing different quantities of calcium demonstrated highly significant direct as well as inverse correlations between medium calcium concentrations and selected chemokines. The regression analyses yielded *r*^2^ values between 0.73 and 0.87 [17] and suggested that extracellular calcium concentration may contribute to modification of peripheral blood mononuclear cell chemokine production. Additionally, a study published by Rossol et al. in 2012 [18] indicated that extracellular calcium stimulated the nod-like receptor protein (NLRP)3 inflammasome, a pattern-recognition receptor in the innate immune system, to increase IL-1β production by monocytes and macrophages. In this situation too, the mediator of the calcium action was the parathyroid CaSR coupled to a G-protein receptor by means of the inositol trisphosphate signaling pathway. This signaling results in an increase of intracellular calcium concentration and inflammasome assembly along with increased caspase 1 production. Thus, while not directly demonstrated to date, given the experimental effect of extracellular calcium on both selected chemokines and cytokines, it is possible that extracellular calcium can either prolong or intensify the inflammatory response to a burn injury, allowing the systemic inflammatory response to sustain itself. Therefore, reduction of circulating ionized calcium by an upregulation of the parathyroid CaSR following burns could potentially dampen or curtail the systemic inflammatory response, while the increase in inflammatory-site calcium may stimulate the inflammasome also via the parathyroid CaSR. Evidence supporting this hypothesis is provided by Jeschke et al. [19], who have demonstrated an increase of burn morbidity in adults at a lower-percentage body surface area burn compared to children. This mechanism can therefore provide a basis for lower burn morbidity and mortality in children as compared to adults. 

**Figure 1:**
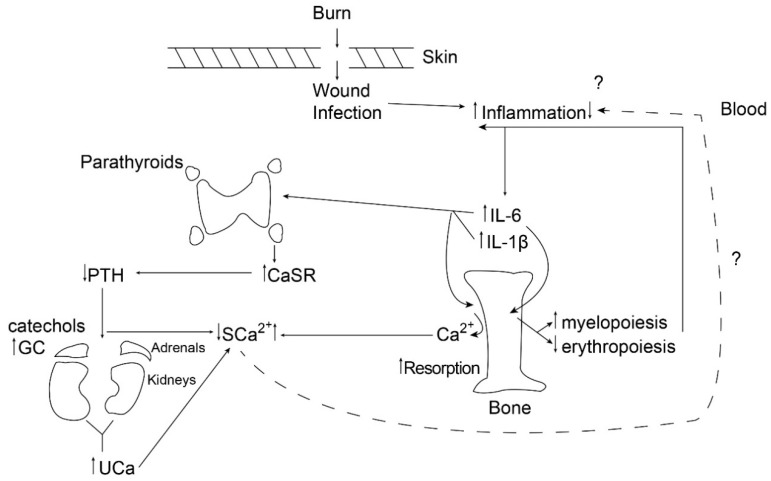
The figure provides a schematic diagram of the chain of events occurring by 24 h following a burn in children, illustrating the balance between liberation of bone calcium by resorption and urinary excretion of calcium following cytokine-mediated upregulation of the parathyroid calcium-sensing receptor. PTH: parathyroid hormone; GC: glucocorticoid; CaSR: calcium-sensing receptor; SCa: serum calcium; UCa: urinary calcium; IL-6: interleukin-6.

It is important to note that the parathyroid CaSR can act as a mediator of inflammation either by upregulating the NLRP3 inflammasome, contributing to increased IL-1 production [18], or by dumping circulating calcium into the urine [13], potentially reducing inflammation. Figure 1 illustrates the dynamics of this process 24 h following a large burn (>40% total body surface area) in children. 

## 3. Bisphosphonates and Calcium

When the antiresorptive bisphosphonate, pamidronate, was given as a single bolus to burned children as part of a randomized, double-blind, controlled study, we found that it immediately curtailed bone resorption, an expected finding [20,21]. We did not anticipate that muscle protein catabolism would also be affected as stable isotope kinetic studies indicated a reduction in muscle protein breakdown and a net positive muscle protein balance in children who received pamidronate as part of the randomized controlled trial, as reported by Borsheim et al. [22]. This finding would suggest that not only were calcium, phosphorus and magnesium not provided for use by activated immune cells [3], muscle breakdown no longer provided amino acid substrates for glucose metabolism by these cells as postulated by Straub [6]. Bisphosphonates work by accumulating in bone matrix. When osteoclasts resorb bone, they take up the bisphosphonate, which inhibits the enzyme farnesyl pyrophosphate synthase (FPS) [23]. This enzyme is part of the cholesterol biosynthesis pathway and as such affects cell membrane composition and signaling. The ultimate result is osteoclast apoptosis. Its reported effect on muscle is likely to be indirect as the studies describing bisphosphonate uptake by muscle suggest that it does not remain for long periods of time [24]. 

Do these findings indicate that bisphosphonates are anti-inflammatory? Certainly no direct evidence exists that they interfere with cytokine production or action. In fact, one of the most common responses to the use of bisphosphonates in adults is the stimulation of γ and δ T cells and the manifestation of a transient flu-like response [25]. Nevertheless, by preventing the products of bone and muscle breakdown from entering the circulation, bisphosphonates may indirectly affect either the duration or intensity of inflammation, but this speculation has yet to be demonstrated. 

## 4. Applicability to Other Inflammatory Conditions

We began with notations of rheumatoid arthritis and inflammatory bowel disease as relatively common conditions in which flares of the inflammatory response promote erosive bone loss. We do not know if the scenario that we described with burn injury pertains to these other conditions as well. The simple answer is no. While Straub [6] postulates the mechanism of action of chronic inflammatory diseases, and while the pathophysiology of burn injury can demonstrate similar features, we do not know if the burns scenario is limited to severe inflammation or whether a lower grade of inflammation will also demonstrate these features. Similarly, we do not know if in other chronic inflammatory disease states, resorption of bone is limited to disease flares or if it also occurs to a lesser degree when inflammation is smoldering. One problem in comparing these conditions is that there is no uniform way of quantitating the inflammatory response across inflammatory conditions. Different cytokines are elevated in different groups of individuals, even between pediatric and adult burn patients [26]. Thus, for example, in severe burn injury, adults and children have different cytokine profiles at different periods post-burn. In the first week post-burn, adults exhibited higher levels of interferon γ, IL-4, IL-6, IL-8, IL-10 and IL-17, and during the second week, IL-1β was higher in children and IL-5 was higher in adults. In rheumatoid arthritis, IL-37 is elevated in serum of affected individuals while almost undetectable in serum of normal individuals [27]. Moreover, IL-37 is also associated with disease activity [27]. In some forms of inflammatory bowel disease, TNF-α and IL-10 are elevated [28]. These are merely examples of the diversity of inflammatory cytokines expressed in different types of inflammatory conditions. 

It may, however, be possible to examine the roles of extracellular calcium, phosphorus and magnesium in different groups of patients with different inflammatory diseases, for example rheumatoid arthritis, inflammatory bowel diseases, systemic lupus and others in various stages of severity in order to try to correlate these cations in the circulation with severity of inflammation. 

## 5. Conclusions

While there is no direct evidence supporting the hypothesis put forward in this paper, I have reviewed the data supporting its plausibility and suggest studies in a variety of inflammatory conditions that might support or refute the relevance of these findings to particular disease states.
